# Treat and Extend Treatment Interval Patterns with Anti-VEGF Therapy in nAMD Patients

**DOI:** 10.3390/vision3030041

**Published:** 2019-08-26

**Authors:** Adrian Skelly, Vladimir Bezlyak, Gerald Liew, Elisabeth Kap, Alexandros Sagkriotis

**Affiliations:** 1 Novartis Pharma AG, 4002 Basel, Switzerland; 2 Westmead Institute for Medical Research, University of Sydney, Westmead, NSW 2145, Australia; 3 IQVIA Commercial GmbH & Co. OHG, 60549 Frankfurt, Germany

**Keywords:** nAMD, anti-VEGF, unlicensed bevacizumab, aflibercept, ranibizumab, age-related macular degeneration, treat and extend

## Abstract

Treat and extend (T&E) is a standard treatment regimen for treating neovascular age-related macular degeneration (nAMD) with anti-vascular endothelial growth factors (anti-VEGFs), but the treatment intervals attained are not well documented. This retrospective, non-comparative, non-randomised study of eyes with nAMD classified treatment interval sequences in a T&E cohort in Australia using Electronic Medical Records (EMR) data. We analysed data from 632 treatment-naïve eyes from 555 patients injected with ranibizumab, aflibercept or unlicensed bevacizumab between January 2012 and June 2016 (mean baseline age 78.0). Eyes were categorised into non-overlapping clusters of interval sequences based on the first 12 months of follow-up. We identified 523 different treatment interval sequences. The largest cluster of 197 (31.5%) eyes attained an 8-week treatment interval before dropping to a shorter frequency, followed by 168 (26.8%) eyes that did not reach or attained a single 8-week interval at the end of the study period. A total of 65 (10.4%) and 83 (13.3%) eyes reached and sustained (≥2 consecutive injection intervals of the same length) an 8 and 12 weekly interval, respectively. This study demonstrates highly individualised treatment patterns in the first year of anti-VEGF therapy in Australia using T&E regimens, with the majority of patients requiring more frequent injections than once every 8 weeks.

## 1. Introduction

Age-related macular degeneration (AMD) is the leading cause of irreversible blindness in developed nations [1]. Vision loss in neovascular or ‘wet’ AMD (nAMD) occurs through the development of choroidal neovascularisation in the macula. These abnormal vessels leak fluid, causing thickening of the central retina and scarring that results in a significant loss of central vision. Vascular endothelial growth factor (VEGF) plays a key pathological role in the formation of these neovascular lesions. For over a decade, the anti-VEGF agents ranibizumab, aflibercept and unlicensed bevacizumab have been used intravitreally to treat patients with nAMD, slowing disease progression and reducing the severity of sight loss [2].

The frequency of intravitreal anti-VEGF injections is an important contributing factor in improving and maintaining visual outcomes [3,4]. In the pivotal ranibizumab and aflibercept studies, patients were treated with monthly injections [5,6,7,8,9]. Treatment schedules have evolved over time since the pivotal studies, as healthcare professionals seek to optimise treatment strategies and reduce the burden of therapy [10,11,12]. PRN (pro re nata; as needed) treatment protocols [13], are used in some regions, but treat and extend (T&E) regimens are now widely established [12]. 

In a T&E regimen, after an initial loading phase of three doses of anti-VEGF given one month apart, patients are injected using monthly intervals until exudative disease activity has resolved. At this point, the interval for the next injection is extended by 1 to 2 weeks up to a maximum of 12 weeks. Upon signs of disease recurrence, evidenced by changes in VA or anatomical parameters such as the presence of intraretinal or subretinal fluid, the treatment interval is shortened by 1 to 2 weeks at a time until the disease is stabilised. 

Thus, T&E is a regimen whereby the anti-VEGF treatment interval is either extended or reduced until the ideal interval for that patient is established, evidenced by stability of vision and/or retinal anatomy. In doing so, treatment interval categories or clusters can emerge, e.g., q4, q6, q8, q12, and clinics avoid the unrealistic burden of monthly visits for all. In short, T&E has gained favour with physicians as the regimen strikes a balance between customisation of treatment to the individual patient’s needs, and a relatively predictable treatment plan, at least in the short term.

T&E was assessed in three prospective, randomised, controlled studies (LUCAS [14,15] and TREX-AMD [16]) and RIVAL [17]. These studies and several other observational studies [18,19,20,21,22] have shown that improvements in visual acuity (VA) and anatomical outcomes, such as decreased central retinal thickness (CRT) and resolution of exudative disease, with a T&E regimen are similar to a monthly regimen, while reducing the injection burden. 

Despite an increasing body of evidence on the clinical efficacy of T&E regimens and injection frequency, limited research has been conducted on injection intervals in real world clinical practice. The only existing evidence is a recent study of an Australian registry (Fight Retinal Blindness [FRB] Registry) of patients with inactive lesions on T&E regimens. According to this non-interventional study, the majority of patients were receiving anti-VEGF therapy at intervals between 4–8 weeks (19.5% of visits occurring once every 4 weeks, 26.9% once every 6 weeks and 28.1% once every 8 weeks); however, as the lesions were inactive when measured, it is not apparent how long after treatment initiation the extended treatment intervals were achieved [23]. Further work is therefore needed to characterise treatment intervals in association to retreatment decisions. 

Selectively identifying interval sequences in T&E presents a challenge due to individualised regimens and different regional treatment practises. One way to overcome this issue is to characterise injection intervals in jurisdictions where T&E is an established regimen. It is known that, in Australia, T&E regimens are used almost exclusively, making it an appropriate setting to capture and study T&E patterns [12,24]. In this study, we observed Electronic Medical Record (EMR) data from Australia to gain insight into treatment interval sequences in T&E regimens from treatment initiation, with a minimum of 12 months follow-up, to improve our understanding of retreatment decisions in real world clinical practice.

## 2. Materials and Methods

### 2.1. Study Design

**Figure 1:**
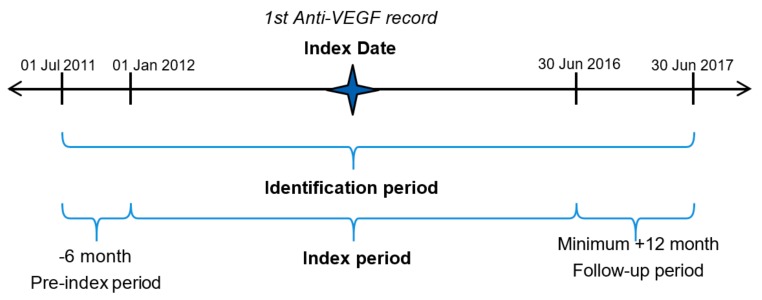
Study setting.

This non-interventional study (NIS) was a retrospective, non-comparative, non-randomised cohort study of patient-eyes with nAMD, treated with intravitreal anti-VEGF injections of ranibizumab, aflibercept or unlicensed bevacizumab during the period between January 2012 and June 2016 (index period; Figure 1). The study was conducted using Electronic Medical Record (EMR; Medisoft) data pooled from four ophthalmology treatment centres in Australia. The study was carried out and reported in accordance with the guidelines for good pharmacoepidemiology practices of the International Society for Pharmacoepidemiology [25], the Strengthening the Reporting of Observational Studies in Epidemiology guidelines (STROBE) [26], and the ethical principles stated in the Declaration of Helsinki [27]. EMR data form a limited dataset in which all patient identifiers are removed, and clinical data are pseudo-anonymised; thus, formal ethics approval or individual patient consent was not required.

### 2.2. Study Participants

**Figure 2:**
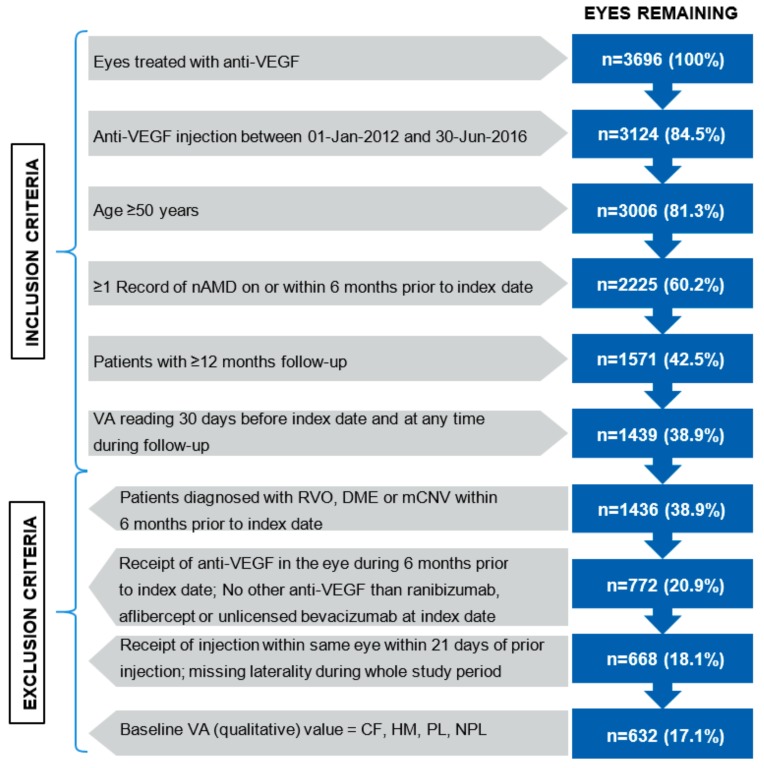
Flowchart describing attrition of the study sample. RVO; retinal vein occlusion, DME; diabetic macular edema, mCNV; myopic choroidal neovascularisation, CF; count fingers, HM; hand motion, PL; light perception, NPL, no light perception.

The study inclusion and exclusion criteria are described in Figure 2. Briefly, eyes were included from patients ≥ 50 years of age, with at least one record of nAMD ≤ 6 months prior to index date (date of the first recorded anti-VEGF injection), and treated with at least one injection of anti-VEGF during the 12 month follow-up period. One or both eyes from a patient could be included. 

Since the date of initial diagnosis could not be confirmed in the EMR network, and/or the patients could be previously treated in a practice outside of the EMR network, it could not be ensured that eyes were treatment naïve; thus, eyes receiving an anti-VEGF injection within 180 days before index date were excluded. The remaining eyes were considered as “treatment-naïve”, as a proxy definition. Eyes were also excluded if they had received an intravitreal injection in the same eye within 21 days of the prior injection. This ensured that laterality was not miscoded and injections were not associated with the same eye; anti-VEGF prescribing information recommends a dosing interval not shorter than 1 month [24]. Patient eyes with a qualitative measure of baseline visual acuity (VA) (i.e., count fingers [CF], hand motion [HM], light perception [PL] or no light perception [NPL]) were excluded, resulting in a loss of 36 patients, considered to have minimal effect on the study outcomes. 

### 2.3. Study Objectives

The primary aim of the study was to explore and define clusters of anti-VEGF treatment intervals in nAMD patient eyes. Secondary objectives were to characterise the VA change from baseline and the time to VA stabilisation. Visual acuity was considered stabilised when VA values remained within a margin of 5 ETDRS letters for at least three consecutive visits. Time to first VA stabilisation was calculated as the number of days from index to the first day of the stabilisation period. Additional secondary endpoints were the number of injections, the number of non-injection visits and the total number of visits for Years 1 and 2.

### 2.4. Outcome Measurements

Patient demographics and clinical characteristics were measured at baseline. Baseline level was defined as the latest record available within the 30 days before and including the index date. The duration of treatment was equal to the number of days between the index injection and the date of last validated update of the database, or discontinuation of treatment, defined as >180 days with no anti-VEGF injections or VA assessment, whichever came first. Eyes were followed up for a minimum of 12 months.

To explore clusters of anti-VEGF treatment interval sequences, treatment intervals were first defined using the same intervals as the FRB! study (of 4, 6, 8, 12, 16 and 20+ weeks; Table 1) [23]. Treatment patterns were determined, based on treatment interval sequence data from individual eyes. A sample set of sequences is given in Supplementary Table S1. Treatment interval sequence data was then categorised into five distinct clusters (Table 2), based on the available data and treatment intervals of interest. There was particular interest in observing q8w injection interval clusters due to recent data suggesting the majority of patients are injected every 4 to 8 weeks in current clinical practice (Clusters 3–5; a maximum of 69 days between two consecutive injections) [10,23].

The intervals were created as follows: Due to exclusion criterion 3, 22 days was selected as the lower limit for intervals. Intervals of greater than 20 weeks were not of interest. However, as patients with no anti-VEGF injections and no VA measurements for >180 days were considered discontinued, the 20-week interval was extended to reflect 20+ weeks. To ensure a seamless transition between 4-week intervals and 8-week intervals, the 8-week intervals were created asymmetrically.

Visual acuity, VA change from baseline and time to VA stabilisation were calculated for the overall cohort and stratified by interval sequence clusters. VA data entered into the EMR are automatically converted to logMAR scores, and thus required conversion to ETDRS letter scores [28]. 

Injection visits were defined as a record of anti-VEGF intravitreal administration; non-injection visits were defined as recorded incidences of optical coherence tomography (OCT), VA, intraocular pressure (IOP) or other assessments without administration of anti-VEGF. The number of injections and visits were calculated per eye for the overall cohort and stratified by interval sequence clusters.

As the study examines treatment in a real-world setting, eyes may not have been assessed on exact visit months; thus, for Months 3, 6, 9 and 12, a window of ±1 month (30 days) was used, while a ±2 month (60 days) window was used for Months 18–24. If more than one VA quantitative measurement was available, the value closest to the month assessed was considered. If two values were equally close, the first value was taken. The value was set as missing if no information was available. 

### 2.5. Statistical Analysis

Sample size was not based on a statistical calculation, since the study was non-comparative and descriptive. Instead, all patient eyes captured in the EMR that met the inclusion and exclusion criteria were considered evaluable. Continuous variables were summarised as the number of observations, means, standard deviations (SDs), medians, interquartile range (IQR) and minimum/maximum values, while categorical variables were summarised using counts and percentages. Two-sided 95% confidence intervals (CIs) were generated in all cases. No missing value imputation was performed. 

## 3. Results

A total of 3696 eyes were captured in the EMR database, of which 632 eyes from 555 patients met the inclusion and exclusion criteria (Figure 2). Six eyes did not have a record of second injection to allow for interval classification. The index drug was ranibizumab for 267 eyes, aflibercept for 155 eyes and unlicensed bevacizumab for 210 eyes. The mean (standard deviation; SD) age of patients whose eyes were included at baseline was 78 (7.8) years and there was a majority of female patients (62.4%). Most patients received unilateral treatment at baseline; 13.9% were treated bilaterally (Table 3). 

**Figure 3:**
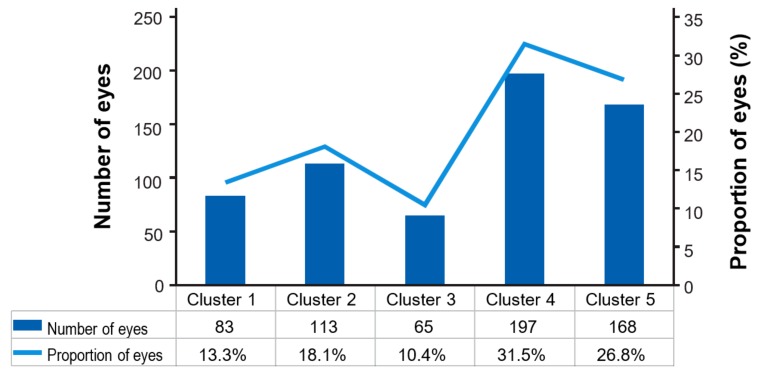
Number and proportion of eyes in the treatment clusters.

A total of 523 different treatment patterns from 632 eyes were identified in the first year of therapy. The majority of patients completed a loading phase (497 eyes, 79%) of ≥3 injections in 90 days post-index. Once all sequences were identified, patient eyes were categorised into five key clusters based on sequence data and treatment intervals of interest (Table 2). The largest category was Cluster 4, which included approximately one third of eyes (197; 31.5%) that reached an 8-week treatment interval and then dropped to a shorter dosing frequency. The second largest category was Cluster 5, comprising 168 (26.8%) eyes that did not reach an 8-week treatment interval or attained a single 8-week interval at the end of the follow-up period. A total of 65 (10.4%) eyes reached and sustained (≥2 intervals) an 8-week treatment interval (Cluster 3), while 83 (13.3%) eyes reached and maintained a 12-week interval (Cluster 1; Figure 3). There were 13 (2%) eyes on fixed 4-weekly dosing, and 118 (18%) eyes reached a 4- to 6-week extension.

The mean (SD) baseline VA of the overall sample was 55.9 (17.1) letters. The highest mean (SD) VA change from baseline in the overall sample was observed at Month 6, with a gain of 6.3 (15.5) letters, followed by a decline to a 3.2 (19.8) letter gain from baseline at Month 24. In the overall cohort, 496 eyes (78.5%) reached stabilised VA, with a median (IQR) of 118.5 (56.5–276.5) days to VA stabilisation (Table 4). 

In Clusters 1–5, baseline age was comparable and ranged from 78.0 to 79.4 years. Baseline VA was also similar across cluster groups, ranging from 53.7 to 57.3 letters, within a margin of 5 ETDRS letters. The largest gain in VA at stabilisation of 8.1 letters was found in Cluster 3, containing eyes that reached and sustained (≥2) q8w dosing intervals without reaching q12w dosing. Eyes in Cluster 1 (includes eyes that reached and remained stable on q12w dosing) exhibited a numerically lower stabilised VA gain of 5.3 letters when compared with the other clusters. Cluster 1 also had a substantially greater percentage of eyes that lost ≥15 letters (stabilised VA loss of 7.2%) when compared with Clusters 2–5 (stabilised VA loss ranging from 1.5 to 4.4%). However, of all the clusters, Cluster 1 also demonstrated the shortest time to VA stabilisation of 92.0 (35.0–346.0) days (Table 4).

**Figure 4:**
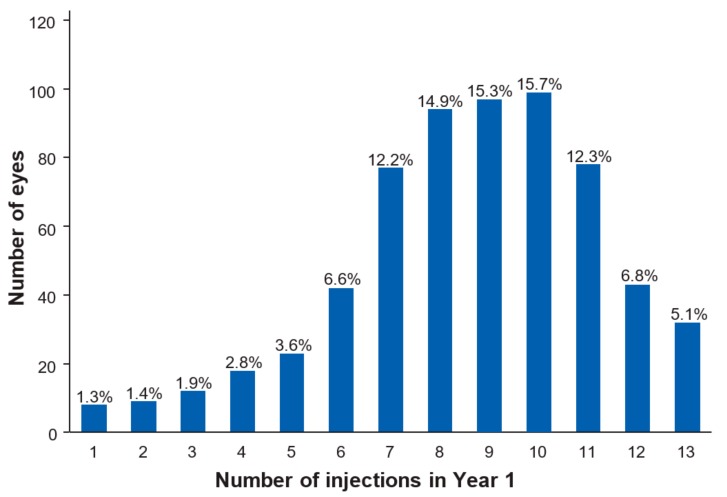
Distribution of total injections received by individual eyes in Year 1.

In Year 1 of follow-up, the mean (SD) number of injections including the loading phase was 8.6 (2.6) and the mean (SD) number of total visits was 9.7 (2.6; Table 5). In Year 2, the mean (SD) number of injections was 4.9 (2.9), and the mean (SD) number of total visits was 5.8 (3.2). Figure 4 shows the distribution of the frequency of injections eyes received in Year 1. The majority of eyes (70.4%) required between 7 and 11 injections. The most common injection frequency was 10 injections in the first year (99 eyes; 15.7%). The highest number of injections given was 13, received by 32 (5.0%) eyes. Injection and visit data stratified by cluster show that from Cluster 1 to 5, the frequency of injections and visits increases, as expected, from 5.7 and 6.9 (Cluster 1; Year 1) to 10.7 and 11.5 (Cluster 5, Year 1). A similar trend is observable for Year 2.

## 4. Discussion

Data from randomised controlled studies show T&E regimens optimise anti-VEGF therapy for patients and overburdened healthcare systems and they have been widely adopted. However, there are limited data on how T&E regimens are adapted for use in the real world. One of the greatest challenges is to identify treatment interval sequences when T&E regimens are highly individualised. Identifying treatment sequence patterns can offer valuable insights into retreatment decisions in the real world and inform future clinical studies to ensure patients’ needs are being met. 

This study uniquely developed clusters based on treatment intervals of interest to categorise these highly individualised treatment sequences. Cluster analysis allows some unification of diverse treatment patterns and could provide key additional insights into retreatment decisions, while being arguably more representative of real-world data when compared with stable treatment interval analysis. In Australia, T&E regimens have been universally adopted thus EMR data from this region may offer insights into T&E treatment strategies in Australia and other jurisdictions that are not possible using data from other regions [24,29].

It is well documented that all patients in Australia who are eligible for anti-VEGF treatment for nAMD are treated using a T&E regimen; however, not all patients follow the protocol, as would be expected in the real world. In this study, EMR treatment pattern data demonstrated the majority of eyes (72%) completed a loading phase of ≥3 doses in the 90 days post-index. This is consistent with 79% of eyes reported in the Fight Retinal Blindness (FRB!) study, which also evaluated T&E regimens in Australia [10]. Beyond the loading phase, there was unexpectedly high variability in treatment patterns, with 523 different treatment sequences reported from 632 eyes in Year 1. Accounting for this observed variability is difficult, as retreatment decisions are taken on a patient-by-patient basis and are not reported in the EMR. Physicians retreat based on clinical evidence of disease reactivation, such as the recurrence of fluid, or decline in vision; the aim of treatment is to maintain an exudation-free macula, but practical considerations in the real world can also influence retreatment strategies. These include different treatment practices in different clinics and real-world factors that influence the timing of retreatment, such as the geographical location of the clinic and ease or difficulty of patient attendance. Patient factors, such as patient comorbidities, patient choice and treatment non-adherence, where the patient or caregiver is not available on the proposed treatment date, may also play a role. 

To characterise treatment patterns, we explored and defined clusters of anti-VEGF treatment interval sequences. Clusters defining treatment around q8w intervals were of particular interest. This study found the majority of eyes required treatment more frequently than once every 8 weeks during the first year of therapy, with 10.4% of eyes reaching and maintaining an 8-week dosing interval. Although approximately 30% of patients reached a q12w treatment interval (Cluster 1 and Cluster 2); fewer than 50% of these patients (and only 13.3% of the overall cohort; Cluster 1) were able to sustain a stable q12w treatment interval. In comparison, data from the FRB! Registry study in Australia, which observed stable treatment intervals, showed that from 4 to 6 months of follow-up, most eyes (59%) had extended treatment to between 5 to 8 weeks. Beyond 6 months, approximately one-third of eyes (21–29%) were treated at intervals of 9 to 15 weeks [10]. Dispensing data from regional pharmacies across Australia suggest that licensed anti-VEGFs are dispensed at approximately 5-weekly intervals by Month 6, and at approximately 8-weekly intervals by Month 12 [30]. Although data from these studies are not directly comparable to the present study—the FRB! study used different time points for interval assessment—there is a broad agreement in the results. In this study, 69% of patient’s eyes (Clusters 3–5) attained at least one q8w injection interval, while 31% of eyes (Clusters 1–2) were treated with at least one q12w injection interval.

VA data for the overall cohort are consistent with results from the FRB! study, where baseline VA was 56.5 letters and peak VA gain at Month 6 corresponded to a gain of 6.4 letters and a subsequent slow decline to 5.3 letters gain at 24 months [10]. In the overall cohort in this study, 496 eyes (78.5%) reached stabilised VA and median (IQR) number of days to VA stabilisation was 118.5 (56.5–276.5) days (approximately 17 weeks). Although the EMR contains limited anatomical data and we cannot directly link VA stabilisation to lesion inactivity in this study, the time to VA stabilisation is similar to a previous real-world FRB! study containing anatomical data in which patients achieved inactivation of lesions by a median (IQR) of 15 (9–29) weeks [31].

Injection and visit frequencies in this study are broadly similar to the FRB! study [10] and dispensing patterns [30] in Australia. Dispensing data can be used as a proxy for injection data, and a recent study found 8.9 units of both ranibizumab and aflibercept were dispensed in the first 12 months of nAMD treatment [32]. Mean numbers of injections in FRB! were slightly lower in Year 1 (7.5 injections) than in the current study [10]. A study of UK and Australian T&E and PRN regimens revealed higher numbers of injections in the first year of follow-up than in the present study (mean number of 9.3 injections) [12]. Differences between the studies could be attributed to differences in treatment strategies in different clinics and/or changes in treatment practice over time. The number of visits compared with the number of injections in the current study are a good reflection of the use of a T&E regimen in clinical practice in the real world, with the majority of clusters demonstrating a difference of <1.0 visit. The slight discrepancy between visits and injections may be due to the measurements being recorded on a different day to the day the patient sees the ophthalmologist, additional unscheduled visits, visits for a different indication (e.g., for the other eye), or a decision at the time of the scheduled visit not to treat.

Analysis of VA and injection visit frequency stratified by cluster sequence can also offer further insights, although the stratified cohorts are small, and any conclusions must be interpreted with caution. Cluster stratification data show that as we move from Clusters 1–5 in each of Years 1 and 2, the number of visits and injections increases, reflecting higher drug utilisation, as would be expected. In Cluster 1, eyes were dosed less frequently than in other clusters and appear to have numerically lower VA outcomes than clusters where dosing is more frequent. However, we cannot rule out that this could also be attributed to reasons such as differences in the patient populations captured in each cluster (though baseline age and VA are comparable). Furthermore, it seems that patients in Cluster 1 had a higher response in the first 6-months post-index, leading to a shorter time until VA stabilisation compared with other clusters. This might have triggered an earlier decision to extend injection intervals, with the consequence of more vision loss by the end of the first year, though interpretation of results in the absence of OCT is difficult. Overall, cluster data indicate that most patients in Australia receive more frequent dosing than once every 8 weeks to maintain visual outcomes.

Several assumptions and study limitations may confound the results. Patients were assumed to be treatment-naïve (by excluding eyes that received any anti-VEGF in the 180 days before the index date) and to be treated according to a T&E protocol, though this information is not recorded in the EMR. As VA data entered into the EMR are automatically converted to logMAR scores, these required conversion back to letter scores, which may have introduced some error. This study does not break down the clusters into the two separate licensed anti-VEGF agents and unlicensed bevacizumab used for nAMD treatment, as the resulting sample size would be too small for robust conclusions. Furthermore, ophthalmologists often switch treatments, which would confound the results. The need for censoring at the time of switch would further reduce the evaluable population. There were also too few patients in the current study to perform a 2-year analysis, though a longer follow-up period would add meaningful insight. 

The limitations of EMR data and real-world evidence have been well documented elsewhere [12,33]. Briefly, as the dataset only includes data from centres using the Medisoft EMR software that consented to share results, patients included in this study may not fully reflect the target population. EMR data also provide information on the drug prescribed not the drug dispensed; though data for number of injections in Year 1 closely resemble dispensing data from another study [30]. Verification of the validity of the diagnosis or study endpoint is not possible from EMR data, reasons for retreatment are not typically disclosed and OCT data are lacking. However, EMR data includes clear diagnoses and transparent outcomes data for individual patients not available in other real-world sources and it is a robust dataset, with highly structured data entry and detailed recording of patient and clinical attributes. Despite limitations, EMR data are considered very representative of real-life clinical care and generalizable to the overall patient population [33]. For the purposes of the study outcomes, EMR data offers an independent and valuable data source that reflects treatment decisions in the clinic [33].

In conclusion, patients treated in a real-world setting under a T&E regimen demonstrate a high degree of variability in their treatment needs, with the majority requiring treatment more frequently than once every 8 weeks in order to maintain vision. The implications of these findings are significant. They suggest that, despite outcomes that are similar to a monthly regimen with a reduced injection burden, a T&E regimen cannot fully address the capacity issues faced by AMD clinics the world over, and innovative solutions are required. One potential solution is the development of more durable and potent anti-VEGF therapies that allow extended injection intervals while maintaining treatment efficacy. 

## Figures and Tables

**Table 1:** Treatment interval classification.

Injection Treatment Pattern	Days from Previous Injection Lower Limit	Days from Previous Injection Upper Limit
4-week interval	22	34
6-week interval	35	48
8-week interval	49	69
12-week interval	70	97
16-week interval	98	125
20+-week interval	126	≥180 *

* ≥180 day treatment interval is possible if the eye has visual acuity measurements without injections.

**Table 2:** Baseline patient characteristics—overall cohort.

Characteristics	Overall Cohort *N* = 555 Patients, *n* = 632 * Eyes
Age in database (patient)	
Mean (SD)	78 (7.8)
Gender (n, %)	
Female	254 (62.4%)
Eyes treated (patient; *n*, %)	
Unilateral	478 (86.1%)
Bilateral	77 (13.9%)
Eyes treated (patient, with laterality specified; *n*, %)	
Left	217 (39.1%)
Right	261 (47.0%)
Both	77 (13.9%)
VA study eye letters	
Mean (SD)	55.9 (17.1)
Central retinal thickness (*n*, %)	289 (45.7%)
Mean (µm, SD)	331 (107)
Year of baseline visit	
2012	214 (33.9%)
2013	174 (27.5%)
2014	141 (22.3%)
2015	82 (13.0%)
2016	21 (3.3%)

* Six eyes did not have a record of second injection to allow for interval calculation and cluster assignment. These patients had multiple visits where VA was determined.

**Table 3:** Cluster classification.

Clusters (*n* = 632 * Eyes)	Description
Cluster 1	Eyes with a record of interval of ≥12 weeks ^§^ that remain on this interval during follow-up visits
Cluster 2	Eyes with a record of interval of ≥12 weeks ^§^ after which a shorter treatment interval occurred
Cluster 3	Eyes with a record of consecutive (≥2) 8-week intervals without a record of a 12-week interval and without a shorter treatment occurring after these intervals
Cluster 4	Eyes with a record of an 8-week interval, after which at least one shorter treatment interval occurs. Subsequent intervals may be of shorter or longer duration
Cluster 5	Eyes with all intervals below 8 weeks or with a record of a single 8-week interval at the end of the study period

* Six eyes did not have a record of second injection to allow for interval calculation and cluster assignment. ^§^ Intervals of 16 to 20+ weeks were treated as a 12-week interval when defining Clusters 1 and 2.

**Table 4:** Visual acuity and change from baseline for overall cohort and clusters.

Characteristics	Overall Cohort *n* = 555 Patients, *n* = 632 * Eyes		Cluster 1 *n* = 79 Patients, *n* = 83 Eyes		Cluster 2 *n* = 107 Patients, *n* = 113 Eyes		Cluster 3 *n* = 63 Patients, *n* = 65 Eyes		Cluster 4 *n* = 182 Patients, *n* = 197 Eyes		Cluster 5 *n* = 154 Patients, *n* = 168 Eyes	
Baseline age (years; SD)	78.0 (7.8)		78.3 (8.5)		78.2 (7.7)		79.4 (7.7)		77.5 (8.1)		78.0 (7.3)	
Baseline VA (letters; n, %)	632 (100.0%)		83 (100%)		113 (100%)		65 (100%)		197 (100%)		168 (100%)	
Mean (SD)	55.9 (17.1)		53.7 (19.6)		57.3 (16.3)		56.3 (14.7)		56.1 (17.0)		54.8 (17.1)	
VA stabilised ^§^ (n, %)	496 (78.5%)		63 (75.9%)		84 (74.3%)		51 (78.5%)		154 (78.2%)		139 (82.7%)	
Median (Q1; Q3) time to VA stabilisation (days)	118.5 (56.5–276.5)		92.0 (35.0–346.0)		196.5 (80.5–341.0)		138.0 (48.0–268.0)		114.0 (56.0–296.0)		94.0 (57.0–223.0)	
Mean (SD) change in VA at stabilisation (letters)	6.7 (15.7)		5.3 (20.7)		6.9 (18.2)		8.1 (15.4)		7.0 (13.5)		6.4 (14.4)	
Mean (SD) VA at stabilisation (letters)	63.4 (16.3)		60.1 (22.0)		65.3 (14.5)		65.2 (14.3)		64.3 (13.9)		61.7 (17.1)	
Loss of letters at stabilisation (n, %)												
≥15 letters	23 (3.6)		6 (7.2)		5 (4.4)		1 (1.5)		6 (3.0)		5 (3.0)	
10–14 letters	26 (4.1)		2 (2.4)		5 (4.4)		3 (4.6)		5 (2.5)		11 (6.5)	
5–9 letters	29 (4.6)		3 (3.6)		7 (6.2)		1 (1.5)		10 (5.1)		8 (4.8)	
1–4 letters	38 (6.0)		4 (4.8)		6 (5.3)		2 (3.1)		11 (5.6)		15 (8.9)	
No change in VA at stabilisation (n, %)	76 (12.0%)		10 (12.0%)		11 (9.7%)		8 (12.3%)		27 (13.7%)		17 (10.1%)	
VA gain at stabilisation (n, %)												
1–4 letters	48 (7.6)		7 (8.4)		5 (4.4)		1 (1.5)		17 (8.6%)		17 (10.1)	
5–9 letters	71 (11.2)		9 (10.8)		9 (8.0)		14 (21.5)		21 (10.7)		17 (10.1)	
10–14 letters	52 (8.2)		5 (6.0)		9 (8.0)		7 (10.8)		21 (10.7)		10 (6.0)	
≥15 letters	133 (21.0)		17 (20.5)		27 (23.9)		14 (21.5)		36 (18.3)		39 (23.2)	
Change in VA from baseline to:	n, %	Mean (SD)	n, %	Mean (SD)	n, %	Mean (SD)	n, %	Mean (SD)	n, %	Mean (SD)	n, %	Mean (SD)
Month 3	544 (86.1)	5.9 (14.8)	71 (85.5)	6.9 (17.4)	82 (72.6)	3.5 (18.5)	63 (96.9)	8.1 (12.0)	181 (91.9)	6.4 (14.2)	142 (84.5	5.6 (12.8)
Month 6	546 (86.4%)	6.3 (15.5)	67 (80.7%)	7.3 (17.4)	88 (77.9%)	4.3 (18.1)	62 (95.4%)	7.2 (13.8)	182 (92.4%)	7.1 (14.7)	141 (83.9%)	6.0 (14.9)
Month 12	505 (79.9%)	5.8 (16.7)	53 (63.9%)	4.7 (17.6)	94 (83.2%)	6.3 (17.1)	52 (80.0%)	6.5 (15.5)	171 (86.8%)	6.7 (15.1)	129 (76.8%)	4.9 (19.0)
Month 24	358 (56.6%)	3.2 (19.8)	40 (48.2%)	4.5 (23.2)	66 (58.4%)	2.1 (21.0)	37 (56.9%)	9.4 (15.2)	116 (58.9%)	3.9 (19.4)	98 (58.3%)	0.4 (19.2)

* Six eyes did not have a record of second injection to allow for interval calculation and cluster assessment. ^§^ VA stabilisation defined as three consecutive visits with VA all within a 5-letter margin of the first (so absolute value of [second VA–first VA] is <2.5 letters, and similarly for the [third VA–first VA]). The date of the first of the three such visits is the stabilisation date.

**Table 5:** Number of visits in the follow-up period for overall cohort and clusters.

Characteristics	Overall cohort *N* = 555 Patients, *n* = 632 * Eyes	Cluster 1 *N* = 79 Patients, *n* = 83 Eyes	Cluster 2 *N* = 107 Patients, *n* = 113 Eyes	Cluster 3 *N* = 63 Patients, *n* = 65 Eyes	Cluster 4 *N* = 182 Patients, *n* = 197 Eyes	Cluster 5 *N* = 154 Patients, *n* = 168 Eyes
Eyes with follow-up in Year 1 (*n*, %)	632 (100.0%)	83 (100.0%)	113 (100.0%)	65 (100.0%)	197 (100.0%)	168 (100.0%)
Number of injections (mean, SD)	8.6 (2.6)	5.7 (2.1)	7.3 (1.6)	8.3 (1.1)	9.2 (1.6)	10.7 (2.4)
Number of visits (mean, SD)	9.7 (2.6)	6.9 (1.7)	8.9 (2.6)	9.0 (1.9)	10.0 (2.3)	11.5 (1.9)
Eyes with follow-up in Year 2 (*n*, %)	632 (100.0%)	83 (100.0%)	113 (100.0%)	65 (100.0%)	197 (100.0%)	168 (100.0%)
Number of injections (mean, SD)	4.9 (2.9)	2.8 (2.1)	4.6 (2.3)	4.7 (1.8)	5.5 (2.9)	5.8 (3.2)
Number of visits (mean, SD)	5.8 (3.2)	3.7 (2.3)	5.9 (30.0)	5.3 (2.4)	6.4 (3.4)	6.5 (3.1)

* Six eyes did not have a record of second injection to allow for interval calculation and cluster assignment.

## Supplementary Materials

The following are available online at https://www.mdpi.com/2411-5150/3/3/41/s1, Table S1: provides examples of treatment interval sequences.
