# Supplementary material for: Treat and Extend Treatment Interval Patterns with Anti-VEGF Therapy in nAMD Patients
Source: Vision (Basel). 2019 Aug 26;3(3):41. doi: 10.3390/vision3030041 (PMC6802800; doi:10.3390/vision3030041)
Supplement: Supplementary file 1 [file vision-03-00041-s001.pdf]

Supplementary table

Table S1 Examples of treatment interval sequences

Interval Sequence (weeks)	n	%
4:4:_8_:_8_:_8_:_8_:__12_:	6	1.3%
4:4:_8_:_8_:_8_:__12_:	7	1.5%
4:_8_:_8_:_8_:_8_:__12_:	5	1.1%
4:_8_:_8_:__12_:__12_:	6	1.3%
__12_:__12_:__12_:___16__:	3	0.6%
4:4:_8_:_8_:__12_:__12_:	4	0.9%
4:_8_:_8_:_8_:__12_:__12_:	5	1.1%
__12_:_8_:__12_:__12_:	5	1.1%
__12_:_8_:_8_:_8_:__12_:	2	0.4%
_8_:_8_:__12_:_8_:__12_:	1	0.2%
